# Ultraminiaturized neural implanted constructs display minimal immunologic response

**DOI:** 10.1016/j.mtbio.2025.101819

**Published:** 2025-04-29

**Authors:** Argyris Spyrou, Mikael Sandell, Rikard Grankvist, Theocharis Nikiforos Iordanidis, Göran Stemme, Staffan Holmin, Niclas Roxhed

**Affiliations:** a Department of Micro and Nanosystems, KTH Royal Institute of Technology, Stockholm, Sweden; b MedTechLabs, Karolinska University Hospital, Bioclinicum, Stockholm, Sweden; c Department of Clinical Neuroscience, Karolinska Institutet, Stockhom, Sweden; d Department of Neuroradiology, Karolinska University Hospital, Stockholm, Sweden

**Keywords:** Biocompatibility, Microimplants, Fabrication, Brain, Constructs

## Abstract

Biocompatibility of medical implants poses a significant challenge in medical technology. Neural implants, integral to curative therapies, initially exhibit efficacy but can lead to unforeseen long-term side effects. The material composition and dimensions of implants are critical factors influencing their biocompatibility within brain tissue. Typically, neural implants are identified as foreign entities by the patient's immune system, triggering persistent inflammation and severe adverse effects. In this study, we investigate the host response in mouse brain tissue of implanted microscale constructs measuring 0.1 × 0.1 × 1 mm^3^ fabricated from common microfabrication materials. Magnetic Resonance Imaging (MRI) analysis reveals rapid recovery of brain parenchyma at 6 week interval post-implantation, accompanied by negligible or mild adverse immune responses during the experimental period. Histological assessments and cell marker stainings targeting astroglia, macrophages, and microglia demonstrate minimal impacts of the microconstructs on mouse brain tissue throughout the 24-week implantation period. Our findings indicate that untethered microimplants of this scale may have potential applications in medical technology and medical treatment for various brain diseases. In summary, this study supports the development of potentially biocompatible brain microimplants that could be useful for the long-term management of chronic brain disorders.

## Highlights

• Biocompatible materials for medical microimplants offer new CNS therapy avenues.
• A 24 week-long investigation suggests microconstruct permanence sans removal.
• Silicon microconstructs elicit low inflammation, showcasing prolonged biocompatibility.
• MRI confirms no blood-brain barrier disruption and minimal brain tissue remodeling.
• Microconstructs trigger localized immune response, hinting at fewer complications.

## Graphical abstract

**Figure:**
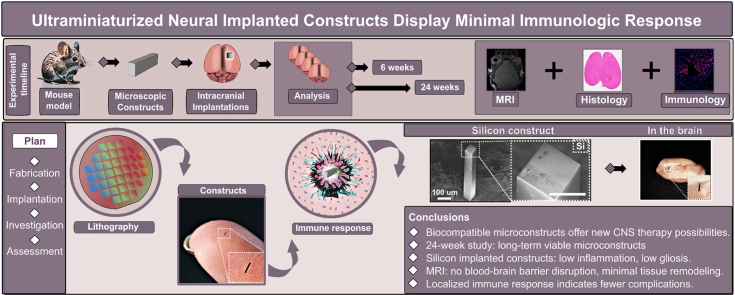

## 1 Introduction

Efficient drug delivery to the restricted confines of the brain stands as a pivotal aspect in the treatment of brain diseases. The Blood-Brain Barrier (BBB), an intricate network of cells including astrocytes and endothelial cells, poses a formidable obstacle to drug delivery into the brain [1]. Its presence precludes numerous potentially effective drugs, such as anticancer agents, from diffusing adequately into the brain parenchyma and accumulating at the desired sites of action [2]. In response to this challenge, the rapid development of central nervous system (CNS) implantable devices for multifaceted applications, including the management of conditions such as Parkinson's disease, depression, and epilepsy [[3], [4], [5]], has revolutionized conventional treatment patterns. However, a significant drawback lies in their biocompatibility [6]. The development of brain implants is inherently limited, and their long-term clinical application entails unclear risks [7]. While there are numerous short-term benefits associated with medical implants, their biocompatibility remains a case of concern due to the potential for long-term side effects in patients [[8], [9], [10]]. A deeper understanding of the mechanisms underlying their biocompatibility is indispensable for the development of more effective and safer novel therapies.

Ensuring safety in neural implants is critical, as their size poses challenges in tissue tolerance, leading to inflammation and glial scar formation due to mechanical imbalances with brain tissue [8,11,12]. Cytotoxicity or inflammation could impair neurological function and reduce treatment efficacy in confined spaces [13]. While larger implants may not be suitable for long-term use [14], smaller, non-tethered small implants are believed to cause less trauma, offering greater precision and functionality [14]. Tethered implants, though useful for continuous monitoring [15], can trigger stronger immune responses and tissue damage due to their invasiveness [14,16]. Non-tethered implants, by minimizing physical interference, offer a more patient-friendly option with reduced chronic inflammation and better immune tolerance.

Non-tethered ultraminiaturized neural implants can offer promising drug delivery possibilities, enabling novel therapeutic strategies. While drugs are key in treating conditions like brain neoplasms [17], their effectiveness often diminishes due to factors like molecular heterogeneity, toxic side effects, or the challenge of crossing the blood-brain barrier (BBB) [2,17]. Direct drug administration to targeted regions could mitigate these issues and improve therapeutic outcomes. Recently developed endovascular techniques, such as the Extroducer™, utilize ultrathin catheters capable of navigating tiny blood vessels (approximately 0.5 mm in diameter) throughout the body and penetrating vessel walls from the inside-out without causing bleeding [18]. This innovative approach enables the precise delivery of drugs, cells, or potentially microimplants into hard-to-reach organs, including the brain. Hence, microimplants of a few hundreds of micrometer in size, loaded with either dry or liquid-based drug formulations, could be utilized for direct implantation into the brain, eliminating the necessity for invasive surgery and subsequent procedures.

External actuation of miniaturized implants presents challenges that some reported studies seem to have overcome, although the device dimensions reported are larger than those in this work [[19], [20], [21]]. A variety of wireless energy transfer methods have been employed to this end, but ultrasound is particularly effective due to its ability to deeply penetrate bodily tissues in a safe manner [22]. Ultrasound for energy transfer deep within the body is mostly used for energy harvesting; however, recent advances have also focused on enhancing drug delivery by triggering thermally responsive hydrogel capsules [20], which can release compounds on demand, activate engineered nanoparticles, or even disrupt cellular barriers [21,23]. These capabilities of ultrasonic energy transfer have not yet been fully utilized for on-demand drug delivery from engineered microdevices. MEMS fabrication technology offers opportunities for developing innovative, biocompatible microimplants precisely engineered to take advantage of ultrasonic interactions for enhanced control and functionality in delivering their payload.

Our study introduces ultraminiaturized, untethered microconstructs (0.1 × 0.1 × 1 mm^3^) to explore their biocompatibility, advancing microimplants as a novel drug delivery system. Unlike larger implants, these microimplants could minimize immune responses by avoiding the friction and inflammation typically associated with larger devices [16,24]. Our objective is to develop these ultraminiaturized non-tethered constructs, evaluate their compatibility with brain tissue over six months, and identify suitable fabrication materials for microimplants. Although metals [13] and borosilicate glass [25] have been proposed as bioinert implant materials, their size limits their applicability. We hypothesize that the small size of these implants may reduce immune response, making them ideal for targeted therapy in hard-to-reach brain regions.

In this study, we investigate biocompatibility of implanted microconstructs measuring 0.1 × 0.1 × 1 mm^3^ in the brains of mice, providing an initial validation for future clinical application in humans. Our investigation evaluates the host response exhibited by commonly used microfabrication materials intended for microscopic neural implants for treating brain diseases. Microconstructs composed of silicon (Si) coated with various common microfabrication materials (e.g., silicon dioxide, parylene, titanium, etc.) exhibited minimal to mild adverse responses in mouse brains. Implanted silicon-based microconstructs induced nominal perilesional inflammation and gliotic scar tissue formation without causing weight loss or other side effects. Our microconstructs elicited immune responses similar to those observed with high-density polyethylene plastic (HDPE), used as a negative control, and displayed contrasting responses compared to the LATEX positive control.

## 2 Materials and methods

### 2.1 Experimental timeline and implantation in mouse brain

**Fig. 1:**
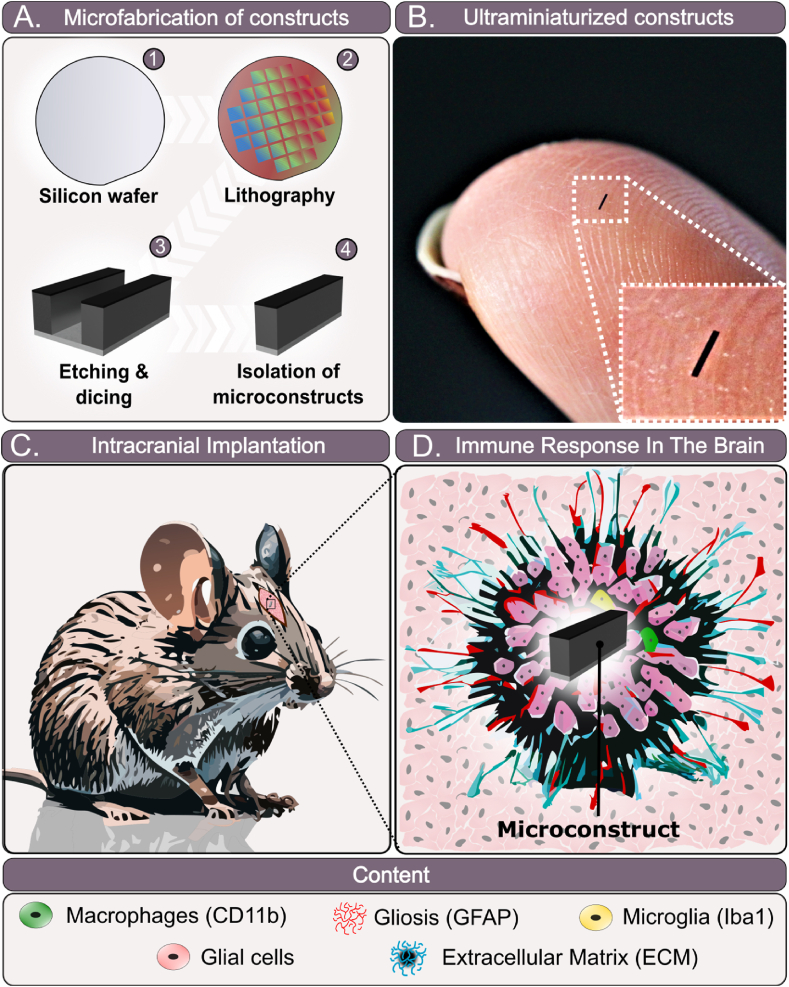
Schematic representation of microfabrication of the microconstructs, experimental design, and immune response in the mouse brain. A) 100 μm × 100 μm × 1 mm silicon based microconstruct structures were fabricated provided with different material coatings and combinations such as PA, Silicon, Silicon dioxide, Titanium, UV-curing glue (adhesive). B) Comparative image of silicon microconstruct structures relative to the index finger". C) Intracranial implantations were conducted using these microconstructs, and the immune response was assessed in the mouse brain through analysis of relevant markers. D) Analysis included staining for astrocytic markers (GFAP) and macrophages (CD11b & Iba1) in the mouse brain to identify potential gliosis and inflammation caused by the implanted microconstructs. (For interpretation of the references to colour in this figure legend, the reader is referred to the Web version of this article.)

**Fig. 2:**
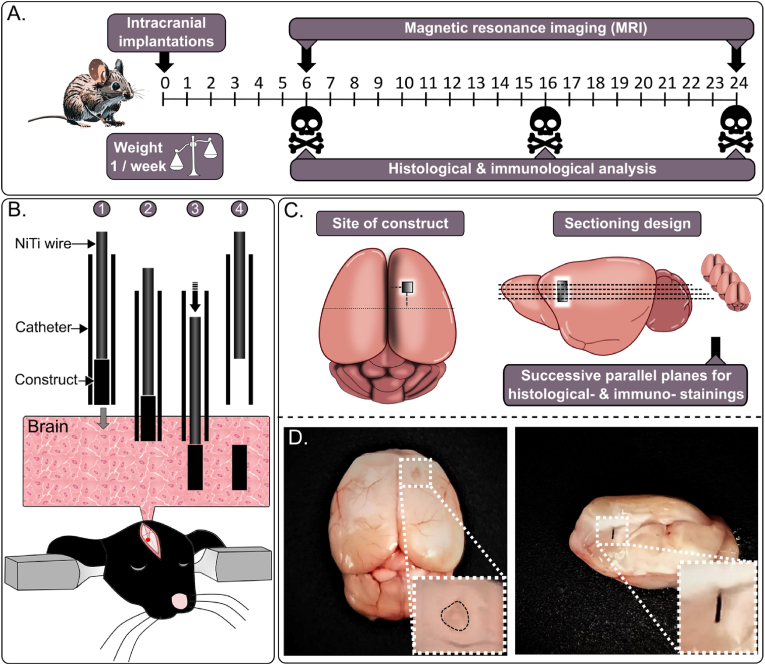
Experimental timeline and microdevice implantation methodology (A) Overview of the experimental setup detailing the microdevice implantation procedure at three timepoints (6, 16, and 24 weeks). The brains from the same mice underwent MRI imaging for a longitudinal study spanning up to 24 weeks. Following imaging, brains were isolated for further analysis, including histological, astrocytic, and macrophage stainings. (B) Detailed depiction of the implantation method: A 2 cm long ultra-thin catheter (ID 280 μm, OD 610 μm) with a nitinol-made wire (D 225 μm) was utilized as a plunger to gently insert the device 3 mm into the brain. (C) The microdevice implantation was conducted at coordinates 1 mm anterior to bregma, 1.5 mm from the midline, and 3 mm below the cranial surface. Illustrations of the dorsal and lateral views of the mouse brain post-implantation are provided to visualize the sectioning approach of isolated mouse brains. (D) Dorsal view of the mouse brain post-microdevice implantation and isolation for further analysis. Additionally, the scheme demonstrates the lateral view of a sectioned mouse brain to clarify the sectioning approach, with the microconstruct visible in the image.

The procedure involved the implantation of microconstructs into the brains of mice (Fig. 1), which were subsequently isolated at designated time points 6, 16, and 24 weeks post-implantation (Fig. 2A). Upon isolation, the brains, along with the microconstructs, were carefully sectioned. After removing the microconstructs, the brains were stained for detailed analysis (Fig. 2C). A longitudinal study was conducted, wherein the same cohort of mice underwent MR imaging from week 6 to week 24. The implantation technique, depicted in Fig. 2, utilized an ultra-thin catheter comprising polytetrafluoroethylene (PTFE) tube with outer and inner diameters of 610 μm and 280 μm, respectively. A nitinol wire, with an outer diameter (OD) of 225 μm, served as a plunger to gently guide the device into the brain, ensuring minimal risk of bleeding or damage to the surrounding tissue (Fig. 2B). The implantation site was located 1 mm anterior to the bregma, 1.5 mm lateral to the midline, and 3 mm below the cranial surface, specifically targeting the frontal cerebral region (Fig. 2C and D). The catheter was carefully inserted into the brain at a speed of 3 mm/s, followed by the NiTi wire, which was used to gently push the microconstructs into the brain at the same speed. The catheter was then removed at 3 mm/s.

### 2.2 Animal studies

All animal studies adhered to the ARRIVE (Animal Research: Reporting of In Vivo Experiments) guidelines and were conducted in compliance with the EU Directive 2010/63/EU and associated guidelines for the care and use of laboratory animals. These experiments were conducted under the ethics permit for animal experimentation with number 6995-2021, as amended by permit number 13839-2022, approved by the Swedish Agricultural Agency. C57Bl/6J female mice aged 6–8 weeks were purchased from Charles-River (Germany). All mouse experiments were conducted in accordance with Swedish legislation and were approved by the Ethics Committee.

### 2.3 Magnetic Resonance Imaging (MRI) of mouse brains after microdevice implantation

A longitudinal study was conducted involving three different mice for each type of microconstruct. The same mice were monitored throughout the experiment and data were collected from week 6 to week 24. Additional mice under the same conditions were sacrificed and brains were isolated for further analysis. Each mouse was imaged by MRI at three different timepoints, at 6, 16, and 24 weeks after the implantation of the devices. MRI was performed at 9.4 T using a scanner with a horizontal bore (Varian, Yarnton, UK). The mice were anesthetized with 4 % isoflurane in a preheated induction chamber, whereafter they were mounted in the supine position with the head in the sensitive region of an actively detuned two-channel phased array coil (Rapid Biomed, Rimpar, Germany). The pose was supported with an in-house designed 3D-printed bed with a bite bar for the incisors, a nosecone and adjustable support structures on the lateral sides of the head. The core body temperature was kept at 37 °C using a feedback controlled warm air system (SA-instruments, Stony Brook, NY, USA). The respiration rate was monitored (Biopac Systems, Inc, Camino, CA, USA) during anesthesia and was kept in the range 50–100 breaths per min by adjusting the isoflurane level in the nose cone. The tail vein was cannulated and the bed with the animal was placed in the bore of the scanner, where the actively tuned birdcage coil (Rapid Biomedical, Rimpar, Germany) was mounted. After scout images, shimming and pulse calibration the brains were characterized through three pulse sequences. Thereafter a Fluid Attenuated Inversion Recovery (FLAIR) dataset was acquired realized using a spin echo sequence prepended by an inversion pulse (tr 4000 ms, ti 1100 ms, te 17.92 ms, FOV 19.2 mm^2^, matrix 256x128, 15 contiguous 0.5 mm thick slices, scan time 9 min 36 s). Lastly, a T1 weighted spin echo datasets were acquired (tr 700 ms, te 17.92 ms, FOV 19.2 mm^2^, matrix 256x128, 15 contiguous 0.5 mm thick slices with two averages resulting in a 2 min 59 s). After the completion of the first T1-sequence 30 μl of 1 M gadobutrol (Gadovist®, Bayer, Berlin, Germany) was injected in the tail vein and the T1-weighted sequence was repeated six consecutive times. The slices were acquired with the read direction in the caudal-rostral direction and the phase encode direction from left to right. The dimensions of lesions depicted on T2-weighted images were determined and calculated utilizing ImageJ software across all subjects within each experimental condition and at all specified time intervals.

### 2.4 Fabrication and scanning electron microscopy (SEM) visualization of implanted microconstructs

**Fig. 3:**
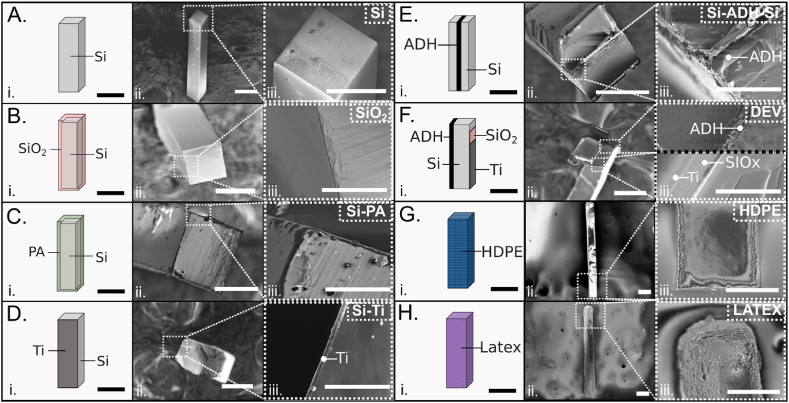
(i) Schematic illustration of the microconstructs fabricated, consisting of various layers shown from top to bottom (A–H): A. Silicon (Si), B. Silicon entirely coated with a 2 μm silicon dioxide layer (SiO_2_), C. Silicon entirely coated with a 2 μm PA layer (Si-PA), D. Silicon with one side coated with 300 nm of titanium (Si-Ti), E. Two 45 μm thick silicon layers bonded with 10 μm thick UV curing adhesive (Si-ADH-Si), F. Silicon with one side coated with 10 μm UV curing adhesive and the opposite side coated with a 2 μm silicon dioxide layer and a 300 nm titanium layer (DEV), G. High-density polyethylene (HDPE), and, H. LATEX (scale bars = 100 μm). (ii) Scanning Electron Microscopy (SEM) images (scale bars = 100 μm) displaying the microconstructs. (iii) Close-up images illustrating the distinct layers of the microconstructs (scale bars = 80 μm). (For interpretation of the references to colour in this figure legend, the reader is referred to the Web version of this article.)

Six distinct types of constructs were fabricated. A. Silicon constructs (Si): Consisting entirely of monocrystalline silicon. B. Silicon dioxide constructs (SiO_2_): Comprising silicon entirely coated with a 2 μm silicon dioxide layer. C. Parylene constructs (Si-PA): Constructed of silicon entirely coated with a 2 μm PA layer. D. Silicon-Titanium constructs (Si-Ti): Featuring silicon with a top layer of titanium (Ti) measuring 300 nm in thickness, applied to only one side of the microstructure. E. Silicon-Adhesive-Silicon constructs (Si-ADH-Si): Formed from silicon components bonded together with a 6 μm layer of EP-OG198-54.3D3 adhesive (non-cytotoxic, UV curing). F. Device construct (DEV): Comprised of silicon with layers of 6 μm EP-OG198-54.3D3 adhesive, 300 nm titanium, and 2 μm silicon dioxide (Fig. 3). SOI wafers with a 100 μm (±10 μm) (Ultrasil, California, US) device layer and an additional 2 μm layer of silicon dioxide on top was used as starting material. Principally, using lithography techniques, specifically, spin coating of 10 μm layer AZ 9260 photoresist (MicroChemicals, Ulm, Germany), defined the construct structures which were then etched 100 μm using Deep Reactive Ion Etching (DRIE, STS ICP, Newport, UK) to the buried oxide layer of the SOI wafer. Subsequently, an HF etch or dicing by Disco DAD 320 (DISCO, Tokyo, Japan) was employed to release the patterned structures from the silicon substrate.

For the silicon dioxide constructs (SiO_2_) (B), the silicon dioxide layer was grown through thermal oxidation of the released silicon structures to obtain a conformal oxide thickness of 2 μm, ensuring complete SiO_2_ coverage (18-h wet oxidation at 1100 °C using a Thermco furnace). Regarding the PA constructs (C), Specialty Coating Systems (SCS) PDS 2010 Labcoter 2 was utilized for deposition of a 2 μm PA layer, resulting in a fully conformal PA coating. The Silicon-Titanium constructs (Si-Ti) (D) were created by evaporating 300 nm titanium before releasing the structures using a Provac PAK 600 Coating System, resulting in constructs with two layers and exposed regions, rather than full coating. In the case of the Silicon-Adhesive-Silicon constructs (Si-ADH-Si) (E), DRIE was used to thin down 50 μm of silicon from the initial 100 μm SOI wafers on two wafers. The now 50 μm SOI wafers were bonded together using 6 μm thick spin-coated EP-OG198-54.3D3 adhesive and structures were thereafter released by dicing; these constructs were not fully coated, as they contain three layers with exposed regions. Lastly, DEV (F) constructs were fabricated using oxidized 100 μm SOI wafers where the silicon device layer was separated using HF, followed by spin-coating of a 6 μm layer of EP-OG198-54.3D3 adhesive. 300 nm titanium was evaporated on the other side through a hard mask leaving areas of silicon dioxide exposed. Finally, the constructs were separated by dicing; these constructs were not fully coated, as they contain four layers with exposed regions. For the fabrication of HDPE (G) and LATEX (H) control constructs, inspired by the findings of Osorio et al. [26], a femtosecond laser (Spirit 1040-4-SHG, Spectra-Physics, Andover, Massachusetts, USA) was used to cut out sections measuring 100 × 1000 μm^2^ from sheets with a thickness of 100 μm of HDPE (1008641A, Bauhaus, Stockholm, Sweden) and LATEX (9000292A, Bauhaus, Stockholm, Sweden). Thus, the HDPE and latex microconstructs are composed entirely of homogeneous HDPE or latex materials, without a silicon core. The laser was focused on the sample surface using an Olympus Plan Achromat RMS4X objective with a numerical aperture of 0.1. The sample was manipulated by a 3-axis linear motorized stage (XMS100, Newport, Rhode Island, USA) with a cutting speed of 100 μm/s. The laser operated at a wavelength of 520 nm with an effective repetition rate of 1 kHz. Optimal cutting power varied for each material and was determined by observing the resulting cuts through the objective using a camera. After fabrication, microconstructs were inspected using scanning electron microscopy (SEM) (Zeiss Ultra 55, Munich, Germany).

### 2.5 Implantation procedure in the brains of mice

Intracranial surgery was conducted to insert a single microconstruct into each mouse brain. Mice were sedated using a mixture of isoflurane and oxygen (0.5–1 L/min oxygen and 5 % isoflurane for anesthesia induction, followed by 1.5–3 % isoflurane for maintenance anesthesia). A hole in the skull was created using a drill. A 225 μm nitinol wire enclosed within a thin PTFE tube (PE10, 427401, BD INTRAMEDIC™, USA) with outer and inner diameters of 610 μm and 280 μm, respectively. The nitinol wire was utilized as a plunger to gently advance the microconstruct into the brain. The microconstructs were stereotactically implanted through the PFTE tube at specific coordinates (1 mm anterior to the bregma, 1.5 mm lateral to the mid-line, and 3 mm below the cranial surface), ensuring precise placement. A control procedure, without a construct, was included in all experiments alongside the other eight construct conditions. Subsequently, the animals were carefully monitored for signs of discomfort or pain over the following 3–4 days.

### 2.6 Processing of isolated brains & histological staining of brain sections

Mice brains were isolated upon reaching the experimental time points of 6 and 24 weeks. For both immunofluorescent and histological (H&E) stainings, mouse brains were fixed in 4 % paraformaldehyde in PBS overnight and subsequently transferred to 70 % ethanol before undergoing paraffin embedding and sectioning (5 μm thickness). For H&E stainings, the brain was first sectioned in the horizontal (axial) plane. Brain sections were initially stained with hematoxylin for 3 min following rehydration, followed by a 10-min immersion in running tap water. Subsequently, the sections were stained for 1 min in eosin solution before being mounted in Pertex Mounting Medium (HistoLab, Askin, Sweden). Whole-slide images were captured using the Olympus SlideView VS200 at the Histological Core facility - HistoCore, Karolinska Institute, Sweden, and were further processed and analyzed using the OlyVIA software.

### 2.7 Indirect immunofluorescent staining of tissue sections and antibodies

Paraffin-embedded tissue sections underwent incubation in a blocking/permeabilization buffer containing 5 % normal goat serum and 0.1 % Triton™ X-100 for 45 min at room temperature. The primary antibodies and their respective dilutions were as follows: Rat GFAP monoclonal antibody (2.2B10) (dilution 1:500, #13–0300, ThermoFisher), Rabbit CD11b monoclonal antibody (EPR1344) (dilution 1:2000, #ab133357, abcam), Mouse IBA1 monoclonal antibody (GT10312) (dilution 1:500, #MA5-27726, ThermoFisher), all in the blocking/permeabilization buffer, incubated overnight at 4 °C. The secondary antibodies and their respective dilutions were as follows: Donkey anti-Rat Alexa Fluor 568 (dilution 1:1000, #A-11077, Invitrogen), Goat anti-Rabbit IgG (H + L) secondary antibody, Alexa Fluor 488 (dilution 1:500, #A-11008, Invitrogen), Goat anti-Mouse IgG (H + L) secondary antibody, Texas Red-X (dilution 1:400, #T-6390, Invitrogen), in PBS for 1 h. Sections were further incubated in DAPI (dilution 1:5000) PBS at room temperature for 15 min before mounting using mounting medium Fluoromount (DAKO). Positive cells in tissue sections were counted both manually (through observation) and automatically (using ImageJ Software) and were scored accordingly. A total of three brains per condition and time point were analyzed for staining.

### 2.8 Statistical analyses

Data were initially assessed for normality using Shapiro-Wilk test. Pairwise comparisons between conditions at same timepoints were performed using independent two-tailed Student's t-tests. Student unpaired *t*-test was used to compare groups for single and multiple comparisons. To determine whether differences existed across all constructs at the same timepoint, one-way analysis of variance (ANOVA) was conducted. The data presented are means ± SE, unless otherwise specified in the figure legends. For each analysis, the p values were represented as follows: NS P > 0.05, ∗P ≤ 0.05, ∗∗P ≤ 0.01, ∗∗∗P ≤ 0.001. Statistical analysis was performed using GraphPad Prism version 8.0 (GraphPad Software, Inc.) and Microsoft Excel.

## 3 Results

### 3.1 Designing microconstructs and investigating their neuroimmune response

A number of brain implanted microconstructs of identical size (0.1 × 0.1 × 1 mm^3^) consisting of different materials and combinations were fabricated, as illustrated in Fig. 1A and B. Following the fabrication, intracranial implantations were performed (Fig. 1C) utilizing these microconstructs. Subsequently, we evaluated the immune response in the mouse brain by analyzing relevant markers (Fig. 1D). We used various techniques, such as MRI, histology and immunofluorescence with cell marker staining, to study comprehensively the mechanisms and neuroimmune responses triggered by the microconstructs at 6 and 24 weeks post-implantation (Fig. 2A–C). Moreover, an in-depth investigation of the perilesional astrocyte (GFAP) and macrophage (CD11b & Iba1) numbers in the mouse brain was undertaken to identify potential occurrences of gliosis and inflammation induced by the microconstructs (Fig. 1D). This multifaceted approach enabled a comprehensive evaluation of the interactions between the brain tissue and the implanted microconstructs, shedding light on their biocompatibility and potential implications for neural interventions.

### 3.2 Visualization and comprehensive characterization of microconstructs

Fig. 3A shows an overview of the microconstructs fabricated for the experiment. All implanted constructs have the same dimensions 0.1 × 0.1 × 1 mm^3^. Visualization of the microconstructs was conducted using SEM, providing detailed information of structure and surface quality: A. Silicon constructs (Si): Comprising solely monocrystalline silicon. B. Silicon oxide constructs (SiO_2_): Consisting of silicon with a 2 μm silicon dioxide layer (Fig. 3B). C. PA constructs: Made from silicon coated with a 2 μm PA layer (Fig. 3C). D. Silicon-Titanium constructs (Si-Ti): Composed of silicon with a 300 nm top titanium (Ti) layer (Fig. 3D). E. Silicon-Adhesive-Silicon constructs (Si-ADH-Si): Formed by silicon parts bonded together with a 6 μm EP-OG198-54.3D3 (non-cytotoxic, UV curing, adhesive) adhesive (Fig. 3E). F. Device construct (DEV): Made of silicon with a 6 μm EP-OG198-54.3D3 adhesive layer, a 300 nm titanium layer, and a 2 μm silicon dioxide layer (Fig. 3F). G. High-density polyethylene (HDPE) microconstructs (Fig. 3G). H. LATEX construct (Fig. 3H): The latter two utilized as pure materials and serving as controls.

By testing various materials our goal was to allow brain cells to be in full contact with the respective materials, enabling us to investigate their effects on the brain (astrocytes, microglia, macrophages, etc.). The core of the different microconstructs was primarily made of silicon for practical reasons, but various materials were coated on top (such as SiO_2_ or PA) to ensure that the cells could recognize and respond to only one material at a time. Since silicon constructs were fabricated and tested alone, we added additional materials such as Titanium (Ti), or Adhesive (ADH), or Silicon oxide (SiO_2_), or combinations of these (DEV), to assess their individual or collective effects on the brain. The materials tested and their combinations were selected because they are widely used in microfabrication techniques and align with the functional and structural requirements of future microimplants.

Ultimately, construct A is made of pure silicon, constructs B and C are fully covered by their respective surface materials, and constructs D, E, and F are composed of various materials which are all exposed to the brain environment. In conclusion, the tissue responses observed for constructs A, B, and C reflect reactions to the individual materials: silicon, silicon oxide, and parylene, respectively. The tissue response to constructs D, E, and F, on the other hand, is a result of the combination of their materials. Our primary goal is to investigate the brain and immune system responses to widely used materials, both individually and in combination, as future microimplants may result from such combinations.

### 3.3 No sign of blood-brain barrier compromise in mice induced by constructs

**Fig. 4:**
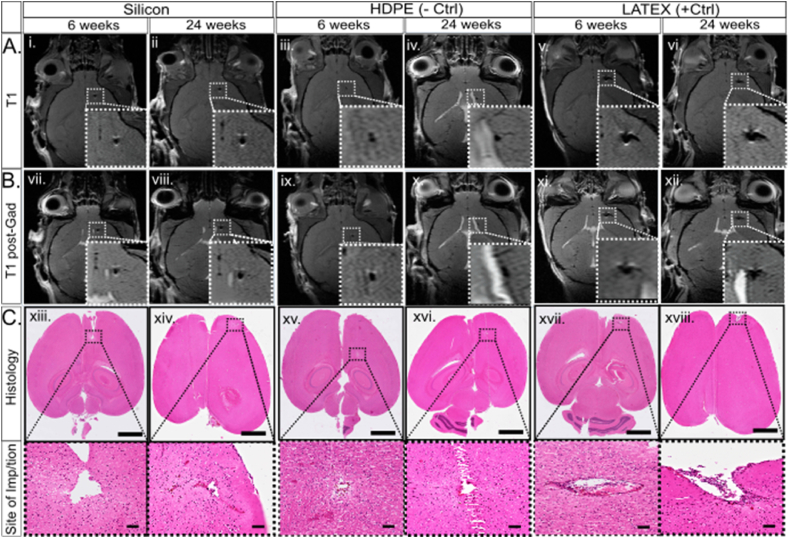
MRI (T1 & T1 post-gadolinium weighted images) and histological (H&E) images illustrating no disruption of the BBB at 6 & 24 weeks post-implantation of the microdevices. T1 (A) & T1 post-gad weighted images (B) display mouse brains implanted with silicon (i, ii, vii, viii), HDPE (iii, iv, ix, x), or LATEX (v, vi, xi, xii) at 6 and 24 weeks post-implantation. H&E staining (C) reveals the implantation site with respective close-up views [whole brain (scale bars = 300 μm) with right side panel of images of the implantation site (scale bars = 100 μm)]. (For interpretation of the references to colour in this figure legend, the reader is referred to the Web version of this article.)

MRI has emerged as a pivotal tool for longitudinal studies in living organisms. In this study, we present brain scans demonstrating the absence of BBB disruption following the implantation of microconstructs in mice. All animals exhibited similar substance defects extending from the posterior frontal lobe to the middle frontal lobe, terminating around the frontal horn of the lateral ventricle on the right side (Fig. 4). This substance defect, referred to as the "lesion" is presumed to represent the microconstruct and/or the canal through which it was inserted. Signal changes, denoted here as perilesional changes, are attributed to the constructs. Analysis of T1-weighted images subsequent to gadolinium contrast agent infusion revealed no signal enhancement in the investigated conditions, indicative of no persistent blood-brain barrier injury (Fig. 4B, T1 post-Gad). This observation suggests that chronic BBB disruption did not occur in any of the conditions, comparable to HDPE (iii, iv, ix, x) and LATEX (v, vi, xi, xii). Brains implanted with silicon (i, ii, vii, viii) (and silicon-based constructs) displayed low signal intensity on T1-weighted images, akin to HDPE (-control), which exhibited normal signal around the lesion. Conversely, mouse brains implanted with LATEX exhibited medium signal intensity in T1-weighted images at both 6- and 24-weeks post-implantation (Fig. 4A, v & vi), indicating that LATEX exerted a mildly adverse effect on the brain. HDPE induced no discernible tissue damage apart from the actual construct lesion, whereas LATEX appeared to induce limited mild gliosis around the lesion center adjacent to the lateral ventricle. LATEX constructs exhibited the most visible changes, while HDPE induced the least. Silicon-based constructs elicited a response classified between the controls, similar to silicon construct. Minor differences were noted between the 6- and 24-week timepoints across all microconstructs, which held no clinical significance during the assessment. However, H&E stainings provided a clearer histological view of the brain, revealing that LATEX caused a relatively wider lesion compared to silicon-based devices and HDPE controls after both 6 and 24 weeks (Fig. 4C), a finding consistent with MR images.

### 3.4 GFAP staining reveals low numbers of perilesional astrocytes induced by silicon-based microconstructs

**Fig. 5:**
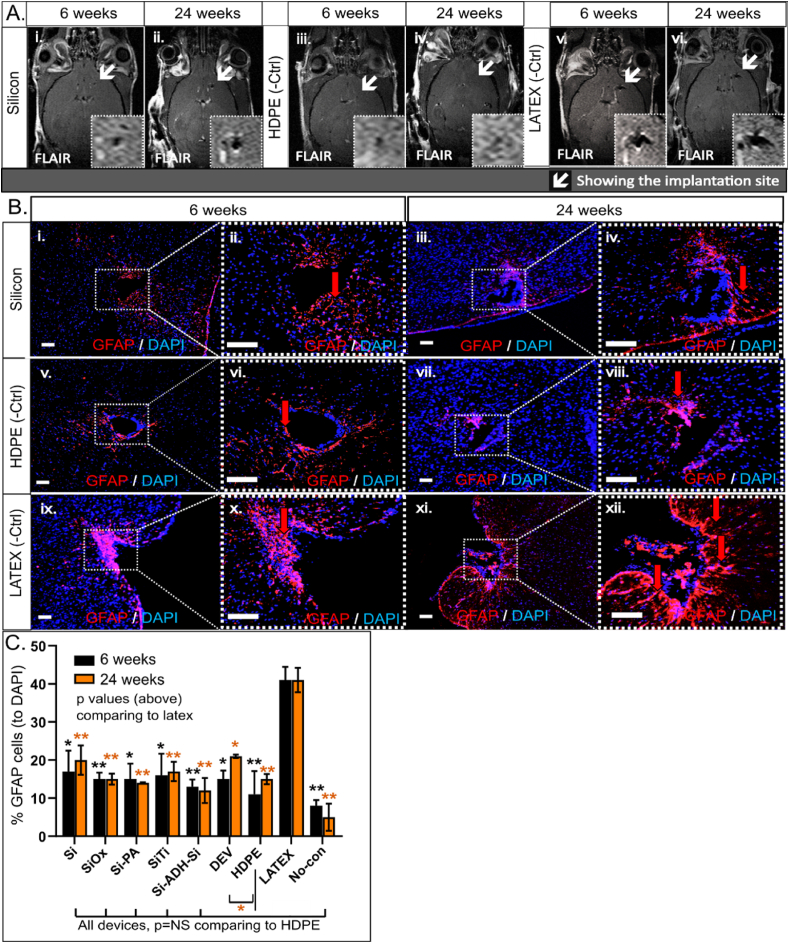
MRI and GFAP staining of mouse brains at 6 & 24 weeks post-implantation of the microdevices. (A) FLAIR images depict signs of gliosis at 6 & 24 weeks in LATEX (v-vi) and silicon-based microconstructs (i-ii). No signs of edema were observed in HDPE-implanted mouse brains (iii-iv). (B) GFAP staining of mouse brains illustrates astrocyte accumulation by each microconstruct and eventual glial scar formation. Mouse brains implanted with silicon-based microdevices (i-iv) exhibited low levels of astrocyte accumulation, comparable to the HDPE condition (v-viii) at both 6 and 24 week timepoints. Astrocyte accumulation was 2.5-fold higher in brains with LATEX microconstructs (ix-xii) (scale bars = 100 μm). (C) Graph displays glial scar formation and assessment of the number of astrocytes. Quantification of GFAP+ cells showed a 2.5-fold increase in astrocyte accumulation around the implantation site of LATEX (+ctrl) compared to the silicon-based microconstructs and HDPE, which exhibited very low levels of astrocyte accumulation around the implantation site (percentage calculated from the visualization of the number of GFAP+ cells normalized to DAPI+ counts, NS=Not significant, ∗ <0.05, ∗∗ <0.01). (For interpretation of the references to colour in this figure legend, the reader is referred to the Web version of this article.)

Scar tissue presents a significant challenge regarding the biocompatibility of microimplants, posing potential hindrances to their functionality. The formation of a dense layer of astrocytes around the implant site can impede microimplant performance, regardless of its intended application. Our objective was to quantify gliosis (scar tissue) formation around the constructs to evaluate microimplant tolerance by the brain parenchyma. MRI serves as a crucial tool for identifying inflammation and assessing gliosis formation and blood-brain barrier disruption. FLAIR images revealed a moderate perilesional signal in brains implanted with LATEX (Fig. 5A–v-vi) compared to the rest of the conditions while brains implanted with HDPE (Fig. 5A, iii-iv) exhibited relatively lower signals. Brains implanted with silicon-based microconstructs (Fig. 5A–i-ii) (not depicted here) showed a mild signal at the perilesional site nearly alike but distinct to LATEX. Notably, HDPE caused no discernible tissue damage aside from the actual construct lesion, whereas LATEX induced mild gliosis around the lesion center adjacent to the lateral ventricle. To further investigate our findings, astrocytic staining was conducted, revealing a high number of perilesional GFAP+ cells around LATEX constructs at both 6 and 24 weeks post-implantation (Fig. 5B, ix-xii). This suggests a significantly increased accumulation of astrocytes (2.5-fold higher) compared to silicon-based microconstructs (∗ for 6 weeks, ∗∗ at 24 weeks) (Fig. 5B, i-iv & S1) and HDPE (∗∗ for 6 weeks, ∗∗ at 24 weeks) (Fig. 5B, v-viii), which displayed only low numbers of astrocytes and no signs of perilesional gliosis. Quantification of GFAP+ cells revealed similar numbers of astrocytes between the 6 and 24-week time points in all conditions, with the all-layer device (DEV) causing higher astrocyte recruitment at 24 weeks compared to 6 weeks (NS) (Fig. 5C). The remaining silicon-based microconstructs exhibited significantly different perilesional GFAP + cell accumulation compared to LATEX at both 6- (∗∗ SiO_2_, ∗ Si-PA, ∗ Si-Ti, ∗∗ Si-ADH-Si, ∗ DEV, ∗ no-construct) and 24-week time points (∗∗ SiO_2_, ∗∗ Si-PA, ∗∗ Si-Ti, ∗∗ Si-ADH-Si, ∗ DEV, ∗∗ no-construct) but showed no significant difference compared to HDPE at both time points (NS), except for the DEV construct, which caused slightly higher astrocyte recruitment at 24 weeks (∗ compared to HDPE).

### 3.5 Microconstructs exhibit lower inflammation levels at 6 weeks and nominal levels at 24 weeks compared to LATEX

**Fig. 6:**
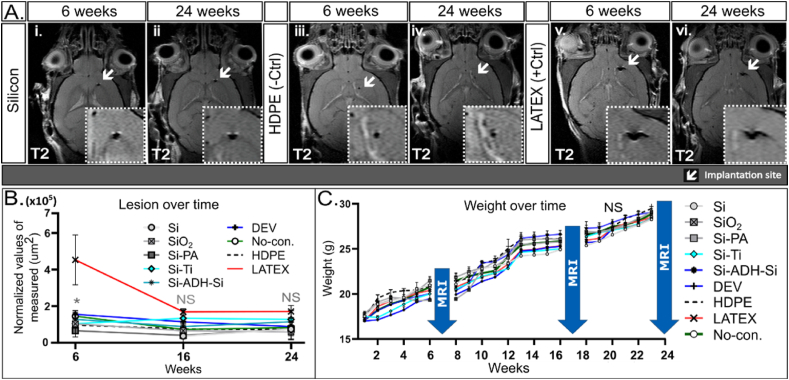
MRI (T2 weighted images) depicting inflammation levels in brains implanted with LATEX (+ctrl) compared to those in silicon-implanted brains at 6 and 24 weeks after microdevice implantation. (A) (i & ii) T2-weighted images at 6 & 24 weeks display low inflammation in silicon-implanted brains. (iii-iv) T2-weighted images at 6 and 24 weeks show no signs of inflammation in HDPE-implanted brains. (v-vi) T2-weighted images at 6 and 24 weeks reveal low signs of inflammation in LATEX compared to HDPE-implanted brains. (B) Line graph representing the size of the lesions plotted as normalized values of the area measured over time from data derived from T2 analysis, indicating LATEX causes the largest lesion diminishing over time compared to any other condition at 6 weeks (∗P < 0.05). However, no significant difference (NS) was observed at 16 or 24 weeks among the groups (C) Line graph illustrating the weight changes of the mice over the 24-week duration of the experiment. The silicon-based microconstructs exhibit similar weight gain over 24 weeks, comparable to HDPE, the negative control, and LATEX, the positive control. MR imaging does not affect the weight of mice, as depicted in the graph. (For interpretation of the references to colour in this figure legend, the reader is referred to the Web version of this article.)

MRI T2 sequences can be used to assess the accumulation of interstitial fluid at the site of interest. Inflammation induced by external materials or injury typically causes fluid accumulation visible on T2 and T2/FLAIR MRI. MRI T2-weighted images depicted mild inflammation signs associated with silicon microconstructs (Fig. 6A/i & ii) and no inflammation with HDPE microconstructs (Fig. 6A/iii & iv) after 6 or 24 weeks, respectively. Examination of both T2 and FLAIR images revealed low signal intensities surrounding the lesions caused by silicon-based devices and HDPE (Fig. 5A/i-iv and Fig. 6A/i & iv), resembling yet differing to LATEX, which exhibited a moderate T2 signal and notably larger lesions at 6 weeks but no significant differences at 24 weeks (Fig. 5A/v & vi and Fig. 6A/v & vi). However, the size of the lesions caused by LATEX was significantly larger (at least 2-fold larger) than those caused by silicon or HDPE (∗P < 0.05) at 6 weeks (Fig. 6B). Therefore, these findings suggest that silicon-based microconstructs do not significantly impact the surrounding tissue (with inflammation comparable to HDPE), unlike LATEX, which exhibits higher inflammation levels and larger lesions.

### 3.6 Absence of significant weight differences in mice across various conditions

Mice implanted with microconstructs underwent careful monitoring, and their weights were measured weekly from baseline (week 0) up to 24 weeks, the termination point of the experiment (Fig. 6C). Remarkably, no weight alterations were observed in any of the mice, irrespective of the microdevice implanted. Throughout the 24-week period, all mice exhibited consistent weight gain, averaging approximately 0.57g per week per mouse (ranging from 0.1g to 1.3g per week), with no significant differences observed among the various conditions (Fig. 6C), similar to the no-construct group. Notably, despite inducing a stronger immune response in mice and causing larger lesions, LATEX did not exert a negative effect on the weight of the mice.

### 3.7 Consistent low numbers of Iba1+ & CD11b+ cells in silicon-based implanted microdevices compared to HDPE (-ctrl) across the experiment

**Fig. 7:**
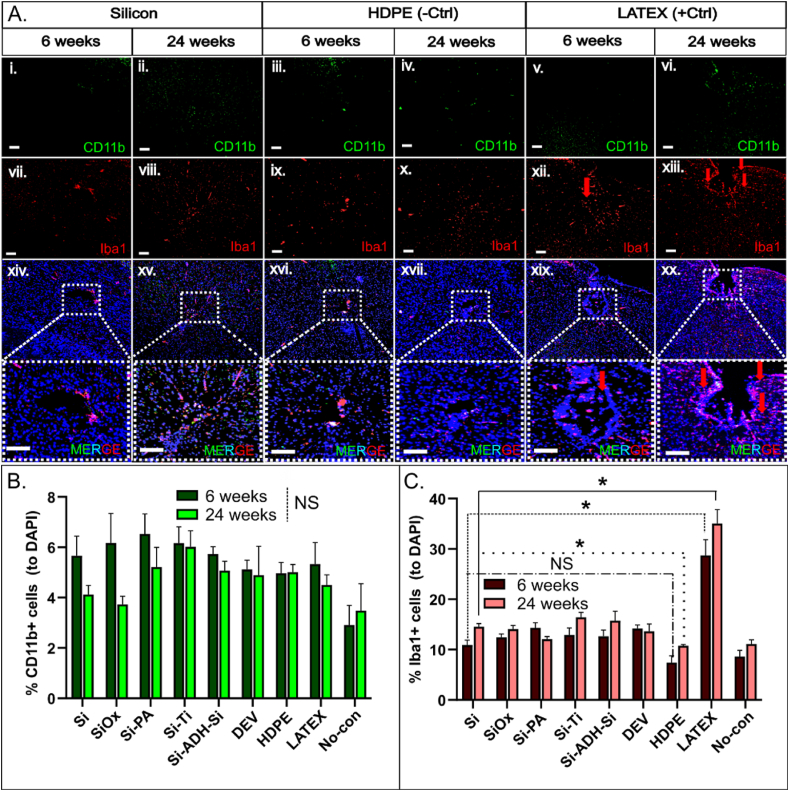
Fluorescent images displaying cells stained with CD11b & Iba1 at 6 & 24 weeks post-implantation of the microdevices (illustrated for silicon, HDPE, & LATEX). (A) Images depict cells stained with the myeloid-origin macrophage marker CD11b, indicating consistent levels of CD11+ macrophages across all devices and timepoints (i-vi). The number of CD11b+ cells remains constant in brains implanted with silicon-based microconstructs, LATEX, or HDPE (scale bars = 100 μm). Fluorescent images of mouse brains stained with the microglial/macrophage marker Iba1 reveal a 3-fold increase in Iba1+ macrophages in brains implanted with LATEX compared to silicon or HDPE (vii-xiii) (scale bars = 100 μm). (B) Bar graph illustrates the number of Iba1 positive cells in mouse brains at 2 different timepoints throughout the experiment (represented for all construct types). (C) Bar graph demonstrates the number of CD11b positive cells in mouse brains at 2 different timepoints throughout the experiment (shown for all construct types, NS=Not significant, ∗ <0.05). (For interpretation of the references to colour in this figure legend, the reader is referred to the Web version of this article.)

Macrophages play a crucial role in initiating, maintaining, and resolving inflammation. To better understand the perilesional response induced by the microconstructs, we conducted staining to identify Iba1+ and CD11b+ cell populations in the isolated brains. Activated microglia and myeloid-derived macrophages are the predominant immune cells involved in both acute and long-term immune responses in the brain. Activated microglia, identified by Iba1+ staining, exhibited increased numbers in brains implanted with LATEX at both 6 and 24 weeks post-implantation (Fig. 7A, xii-xiii), as anticipated. Remarkably, none of the silicon-based microconstructs caused high numbers of Iba1+ cells and showed levels of activation slightly higher (though not significant) to those induced by HDPE (-ctrl) (S3A-F, ii & vi). In contrast, LATEX constructs demonstrated a 2.5-fold increase in activated microglia (Iba1+ cells) compared to most of the silicon-based devices, with HDPE and no-construct conditions inducing the least immune response at both 6 and 24 weeks post-implantation (Fig. 7C & S3). Surprisingly, CD11b+ myeloid-derived macrophages displayed consistent and uniform numbers across all conditions, including controls, with no significant changes observed at either the 6 or 24-week time points (Fig. 7B & S3).

## 4 Discussion

Malignant brain tumors, neurodegenerative diseases, epilepsy, and other neurocognitive disorders require more effective treatment strategies for improved patient outcomes. A key challenge is achieving successful drug delivery into the central nervous system (CNS), as the blood-brain barrier (BBB) and other limitations hinder the clinical efficacy of many drugs despite their promising *in vitro* results [[27], [28], [29]]. Microimplants offer a potential strategy for CNS drug delivery, but concerns remain about their long-term biocompatibility [30], as they can cause complications such as scar tissue formation and adjacent toxicity due to their size or material composition [31,32]. In this study, we evaluate the immune response to common microfabrication materials used in microscopic, non-tethered neural constructs (Fig. 1). Our findings demonstrate that silicon-based neural microconstructs evoke minimal to negligible immune responses in mouse brains, providing a foundation for the development of novel biocompatible brain microimplants for medical applications.

Our 24-week investigation assesses the long-term tolerability of ultraminiaturized (100 μm × 100 μm × 1 mm) neural microconstructs in mouse brains, focusing on their potential for permanent deposition of microimplants without removal. However, significant tissue remodeling and immune infiltration typically occur [30], influenced by implant size, material, density, and stiffness [14,24,33]. Additionally, wireless implants, with short-range mobility, induce less friction and damage compared to tethered counterparts [16,30]. Beyond biocompatibility, drug-loaded microimplants may potentially offer precise, localized drug delivery, minimizing systemic side effects. Despite their small size, they can carry up to 4 μg of medication, achieving peritumoral concentrations comparable to oral chemotherapy, such as temozolomide [34,35] and doxorubicin [36,37]. While drug load capacity is a limitation, multiple implants could enhance therapeutic effects, providing a minimally invasive, targeted alternative for CNS and PNS treatments [38,39].

Acute inflammation, if uncontrolled, can lead to tissue necrosis and scar formation. Triggered by tissue damage or infection, it plays a key role in the foreign body response (FBR), which disrupts tissue homeostasis upon implant insertion [40,41]. FBR may accelerate material degradation by increasing immune cell activity or induce ECM remodeling, causing scar formation or implant displacement [20,23,42]. 9.4T MRI was selected as a precise method for conducting longitudinal studies to monitor inflammation and scarring in living organisms without adverse effects (Fig. 2). The regulated immune response is primarily beneficial, although it can become destructive if not properly balanced [43]. MRI provided detailed visualization of the entire brain tissue and the specifics of the perilesional sites following microconstruct implantation, revealing no disruption of the BBB, as confirmed by pre- and post-gadolinium T1 weighted images (Fig. 4). Neither our implantation technique (Fig. 2B) nor the implanted constructs themselves (Fig. 3) caused any discernible damage to the brain. However, brains implanted with LATEX (+control) exhibited a lesion approximately 2.5 times larger than those with silicon microconstructs and HDPE at the 6-week time point, with a reduction in lesion size by LATEX observed at 16 weeks (Fig. 6B). This finding aligns with the study by Osorio et al., which demonstrated that both HDPE and LATEX initially induce acute inflammation when implanted in the flanks of mice, although inflammation induced by HDPE resolves more quickly compared to the chronic inflammation and scar tissue induced by LATEX [26]. Although HDPE has been shown to be highly bioinert, some foreign body response (FBR) in the surrounding tissue is unavoidable due to its insertion into the tissue. Thus, localized early inflammation likely explains why LATEX may have caused larger lesions around the implantation point but not in other conditions. This finding contradicts the findings of Stiller et al., who demonstrated that the severity of the immune response caused by a device is highly correlated with device stiffness [33]. In our case, this correlation does not necessarily apply, as the softer the microconstruct (with LATEX being the softest), the stronger the immune response. Nonetheless, we believe that the material (LATEX) itself has a negative impact on the immune response, outweighing its size parameter [44,45]. Similarly, the sharpness of the constructs does not appear to play a significant role in microscopic implants. While sharp implant corners can increase the risk of tissue damage during implantation, particularly in the case of larger devices [46], the small size of our implanted microconstructs minimizes this concern. HDPE and LATEX exhibited opposite immune responses in the brain, despite being the same size. All silicon-based microconstructs caused significantly smaller lesions than LATEX, with HDPE and no-construct conditions showing the smallest lesions (Fig. 6, Fig. 5D). Histological analysis confirmed that LATEX caused larger lesions with more immune and astrocytic cell recruitment. Brain MRI showed no effect on residual brain parenchyma, indicating that the response remained localized. This is evident from the strong immune response elicited by the soft LATEX compared to the hard, sharp-edged silicon. Nevertheless, medical devices must meet strict safety standards, and smoothing implant edges can further enhance biocompatibility, reduce complications, and improve clinical outcomes.

We aimed to investigate the inflammatory status around the implanted microconstructs at two time points, 6 and 24 weeks, as inflammation is a key contributor to tissue damage and cell accumulation. T2-weighted imaging showed mild fluid accumulation around LATEX constructs (Fig. 6B), with histology confirming greater perilesional cell accumulation and larger lesions compared to other microconstructs (Fig. 4). Other constructs, such as Si-ADH-Si and DEV, displayed higher T2 signal intensity, suggesting a stronger immune response when multiple materials are present (Supplementary Fig. 3). Staining for Iba1+ microglia revealed increased activation around LATEX constructs at both time points, with a 2.5-fold higher microglial response at 24 weeks (Fig. 7C). In contrast, silicon-based constructs, like HDPE, exhibited low Iba1+ cell counts, supporting their biocompatibility. CD11b+ macrophages remained consistent across all conditions, with no significant changes (Fig. 7D).

Myeloid-derived macrophages are typically observed in cases of BBB disruption (e.g., traumatic brain injury, brain tumors, irradiation) but are rarely seen in other circumstances [47,48]. Under steady-state conditions, bone marrow-derived myeloid progenitors lack access to the central nervous system (CNS) and thus do not contribute to the tissue microglial response [49]. However, in experimental models of neuro-inflammation, myeloid monocytes can infiltrate the CNS and contribute to tissue inflammation by exhibiting macrophage behavior. Nevertheless, these phenotypes typically diminish rapidly and depend heavily on the cause of inflammation [50]. In our study, CD11b+ myeloid-derived macrophages did not infiltrate the brain parenchyma at 6 or 24 weeks after implantation. This observation may be primarily explained by the rapid reduction in inflammation before 6 weeks or by the presence of insufficient inflammation in the perilesional region, which failed to trigger a systemic response. Analysis at an earlier time point would not have resolved this issue, as it would have been impossible to differentiate inflammation resulting from the implantation of the microconstruct itself. In conclusion, this suggests a more localized immune response induced by LATEX microconstructs compared to silicon-based microconstructs. Nonetheless, this response led to microglial activation without involvement of CD11b cells in a broader systemic immune response.

A crucial limitation of medical microimplants is undeniably the formation of fibrous tissue, particularly glial scar formation in our case. This could impede the ability to locate, control, and manipulate, emerging microimplants as needed for subsequent interventions. The primary component of glial tissue is the accumulation of astrocytes around the lesion following injury or inflammation. MRI imaging (FLAIR/T2) provided a clue by revealing signs of scar tissue around brain lesions caused by implanted silicon-based microconstructs, a finding similar to that observed with LATEX, which induced mild lesions and potential glial scar formation. Specifically, we then investigated the accumulation of astrocytes around the lesion and the formation of gliosis through cell staining to corroborate the MRI findings. GFAP staining, which marks astrocytes, was performed for all conditions, and LATEX was found to induce the highest number of astrocytes around the lesion (with similar levels observed at 6 and 24 weeks after implantation), explaining the thick cell layer (Fig. 4). These findings are consistent with Kozai et al., who demonstrated an immediate microglial reaction to microelectrode implantation and gliosis formation within 24 h [51]. However, none of our other implanted constructs elicited such astrocyte recruitment, with the no-construct and HDPE devices resulting in the lowest numbers of astrocytes around the lesions. Consequently, there was no significant glial tissue formation in the perilesional site caused by silicon-based microconstructs, consistent with Dabbour et al. [13], where no gliosis was observed. However, this contrasts with findings in other neural implantation models where prominent glial scar tissue was observed [24,52]. Similarly, Prodanov and Delbeke have suggested that implants with a density closer to that of brain tissue will exhibit less glial scarring [30]. While our implanted constructs showed minimal astrocyte recruitment, studies indicate that SiO_2_ nanoparticles can be toxic to astrocytes *in vitro*, causing morphological changes and alterations in protein structures due to their small size, leading to oxidative stress and protein misfolding [53,54]. In contrast, the optimized design of our microconstructs enables close integration with brain tissue, enhancing their effectiveness. The solubility of implant materials though can, in such cases, affect the immune and tissue response, depending on how they are created or deposited onto a surface. Silicon oxide can be produced by chemical vapor deposition (CVD) or plasma-enhanced CVD (PECVD), which may result in different microstructures and densities, making them soluble within hours [55] However, we created silicon oxide through thermal oxidation of silicon wafers, which is highly stable and has a significantly lower dissolution rate. This is significant, as we demonstrate that microimplants made from these materials will induce minimal astrocyte accumulation in brain tissue, thereby reducing scar tissue formation, which could otherwise impair implant functionality and longevity.

Our study underscores the significance of implant size, material properties, and a comprehensive approach to implant design to mitigate the immune response and promote long-term functionality. The implanted silicon-based microconstructs discussed in this study exhibit several advantages over other materials in terms of their immune response. These microconstructs consistently produced significantly smaller lesions, a key indicator of a less aggressive immune response. This observation, shown by MRI analysis, is particularly crucial as it challenges the conventional knowledge that implant stiffness directly correlates with the severity of the immune reaction. We demonstrated that LATEX, despite being the softest material used, triggered the most robust immune response [26]. This emphasizes that material properties beyond stiffness play a crucial role in determining biocompatibility. Specifically, the chemical composition of LATEX likely contributes to the heightened immune response. This further support this concept, highlighting that some materials, such as HDPE, are inherently bioinert, explaining the minimal lesion size observed with HDPE constructs, which is comparable to brains without constructs. The test implantation, without the material (no construct), showed no significant immune response or tissue damage. MRI and histology revealed the smallest lesion size (Fig. 6B) and minimal gliosis (GFAP staining), even lower than the HDPE negative control (Fig. 5C). Perilesional tissue remained unaffected, and the blood-brain barrier was intact. Inflammation (Fig. 7B and C) was similar to the HDPE condition, with comparable levels of CD11b+ and Iba1+ cells. The procedure itself had negligible tissue penetration effects, suggesting observed impacts in other conditions were due to the implanted constructs, confirming no inherent damage or stress to the brain tissue. In contrast, the silicon-based microconstructs consistently exhibited low T2 signal intensity, suggesting minimal fluid accumulation and indicating a localized, less intense immune response. This localized response is further supported by the absence of CD11b+ myeloid-derived macrophages in the brain parenchyma, indicating that the blood-brain barrier remained intact. Larger implants and those with multiple protruding elements tend to produce more bleed damage and subsequent inflammation [[56], [57], [58]]. The silicon-based microconstructs, being small and non-tethered, likely minimize such damage. Furthermore, factors like implant density and the ability to flex with the brain's micromotions can influence the severity of the tissue response [13,[58], [59], [60]]. Additionally, as porosity enhances tissue integration and immune compatibility [61,62], it aids the development of biocompatible microimplants [63]. Similarly, integrating bioresorbable materials may reduce scar tissue formation, improving drug delivery techniques [64,65]. These implants naturally degrade, minimizing chronic inflammation and eliminating the need for removal surgeries [64,66]. While our microconstructs already show long-term tolerance, incorporating porosity and biodegradability could further enhance CNS therapy safety and efficacy by reducing immune responses and optimizing drug delivery. Combined with their ultra-small size, this approach improves brain implant integration. However, careful material selection is essential, balancing biodegradable and non-biodegradable components like silicon. While our investigation does not directly address these issues, the minimal tissue response observed by the silicon-based microconstructs suggests that their small size plays a critical role in minimizing their impact on the brain. These materials can be engineered to match the mechanical properties of brain tissue, reducing strain and potentially minimizing the immune response [58]. However, the study demonstrates that carefully designed silicon-based microconstructs can achieve comparable biocompatibility without the need for additional coatings. Translating findings from animal models to human clinical applications presents various complexities. Additionally, while the study emphasizes minimizing immune responses, other crucial factors, such as the implant's ability to deliver drugs over time, are also essential for its clinical success.

Future applications will enable us to avoid any dilemmas regarding glial scar formation, given that the devices are composed of these materials and are of this size. Additionally, none of the microconstructs resulted in adverse effects on the mice, particularly with regard to mouse weight. Throughout the 24-week period, all mice exhibited no significant weight loss or negative physical or behavioral signs. While LATEX, serving as a positive control, may have induced a considerable reaction, the mice did not exhibit any obvious effects during the 24-week period.

## 5 Conclusions

Our study represents an exploratory effort into the host response of implanted microconstructs made from commonly used microfabrication materials relevant for medical applications. Through thorough examination, we provide evidence supporting the viability of untethered ultraminiaturized implants, particularly as integral components of biocompatible brain microdevices designed for sustained therapeutic interventions. The significance of our investigation extends beyond our current scope, providing insights into the developing area of neurotechnology. The potential for minimally invasive procedures and targeted drug delivery introduces a big change in the approach to long-term treatment modalities, offering possibilities for enhanced patient care and management of chronic conditions. In essence, our study provides an indication for the development and implementation of microscopic biocompatible brain microimplants in medical practice. Ultraminiaturized constructs induce mild and potentially tolerable host responses, at least in mice where their relative size is much larger than in humans. By elucidating the compatibility of microfabrication materials at the microscale level, we show that this is a potential route for medical device technologies, paving the way for innovative solutions that have the potential to change therapeutic interventions for years to come.

## CRediT authorship contribution statement

**Argyris Spyrou:** Writing – review & editing, Writing – original draft, Visualization, Validation, Supervision, Software, Resources, Methodology, Investigation, Formal analysis, Data curation, Conceptualization. **Mikael Sandell:** Writing – review & editing, Writing – original draft, Validation, Software, Formal analysis. **Rikard Grankvist:** Writing – review & editing, Writing – original draft, Visualization, Validation, Software, Investigation, Formal analysis, Data curation. **Theocharis Nikiforos Iordanidis:** Writing – review & editing, Writing – original draft, Visualization, Validation, Investigation, Formal analysis. **Göran Stemme:** Writing – review & editing, Writing – original draft, Resources, Project administration. **Staffan Holmin:** Writing – review & editing, Project administration. **Niclas Roxhed:** Writing – review & editing, Writing – original draft, Visualization, Supervision, Resources, Project administration, Funding acquisition, Data curation, Conceptualization.

## Declaration of generative AI and AI-assisted technologies in the writing process

During the preparation of this work the author(s) used ChatGPT v3.5 to grammatically refine and polish the existing text for grammatical accuracy. After using this tool/service, the author(s) reviewed and edited the content as needed and take(s) full responsibility for the content of the publication.

## Funding

This research was generously funded by MedTechLabs, a strategic collaboration between Karolinska Institutet (KI), KTH Royal Institute of Technology, and Region Stockholm, and we gratefully acknowledge their vital support in enabling this work.

## Declaration of competing interest

The authors declare the following financial interests/personal relationships which may be considered as potential competing interests: Staffan Holmin (SH) and Rikard Grankvist (RG) reports a relationship with SmartCella Holding AB that includes: equity or stocks. Staffan Holmin (SH) and Rikard Grankvist (RG) have financial intererest in the company SmartCella Holding AB that produces the Extroducer technology mentioned in the introduction. No other potential conflicts of interest were disclosed by the authors. If there are other authors, they declare that they have no known competing financial interests or personal relationships that could have appeared to influence the work reported in this paper.

## Data Availability

Data will be made available on request.
