# Supplementary material for: Ultraminiaturized neural implanted constructs display minimal immunologic response
Source: Mater Today Bio. 2025 Apr 29;32:101819. doi: 10.1016/j.mtbio.2025.101819 (PMC12088817; doi:10.1016/j.mtbio.2025.101819)
Supplement: Multimedia component 1 [file mmc1.docx]

**Supplementary figures**


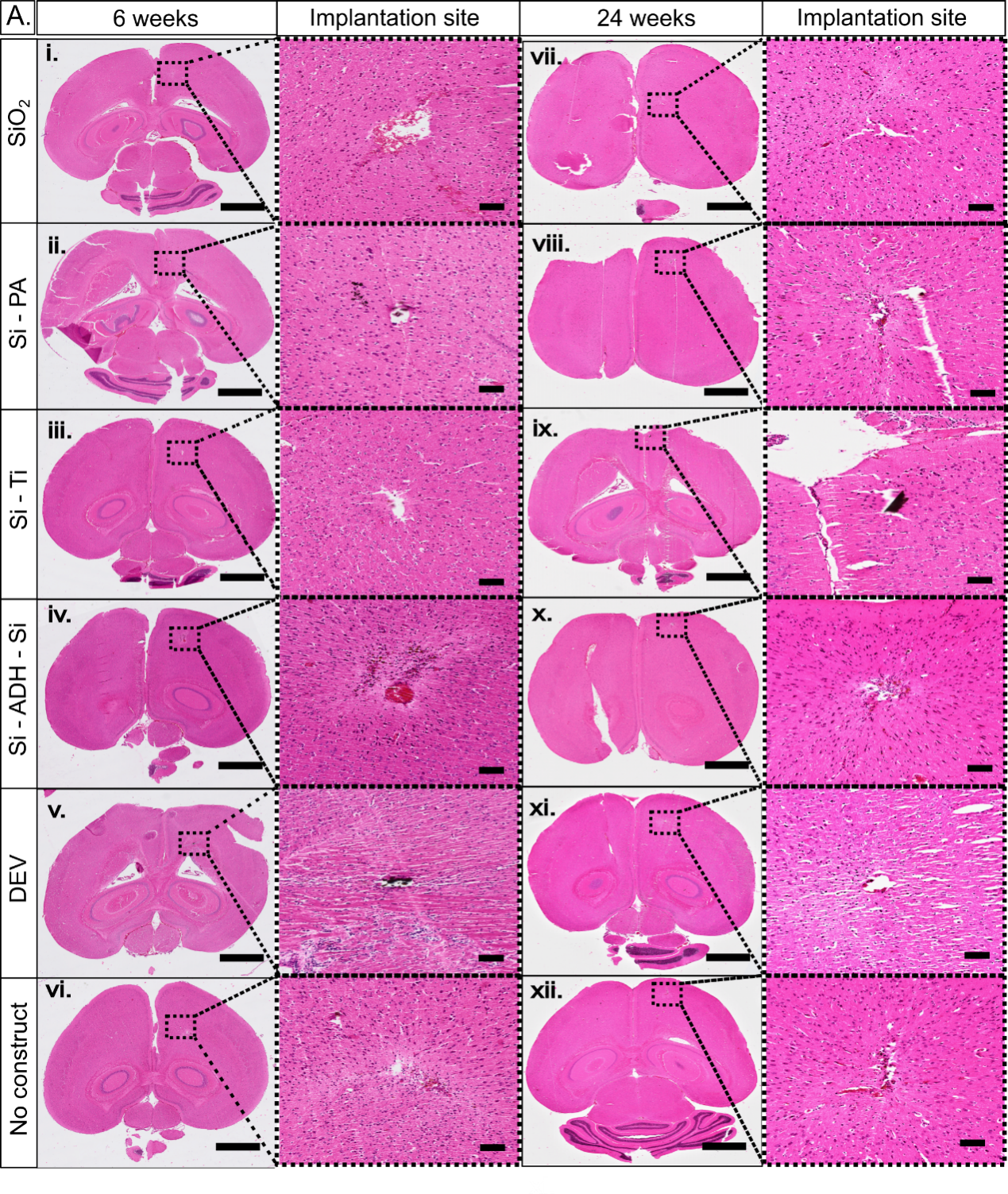


**Figure S1**. H&E staining images (xiii-xviii) depicting the implantation site, including whole brain views (scale bars = 300 µm) and close-up images (scale bars = 100 μm). The images represent various conditions at 6 weeks (i-vi) and 24 weeks (vii-xii) post-implantation of the microconstructs. The microconstructs shown at 6 & 24 weeks respectively include: Silicon oxide (i & vii), silicon coated with a PA layer (ii & viii), silicon with one-side titanium coating (iii-ix), two silicon parts glued with UV curing adhesive (iv & x), silicon with one-side coated with UV curing adhesive and the opposite side coated with silicon oxide plus a titanium layer (v & xi), and a blank sample with no construct (vi & xii).


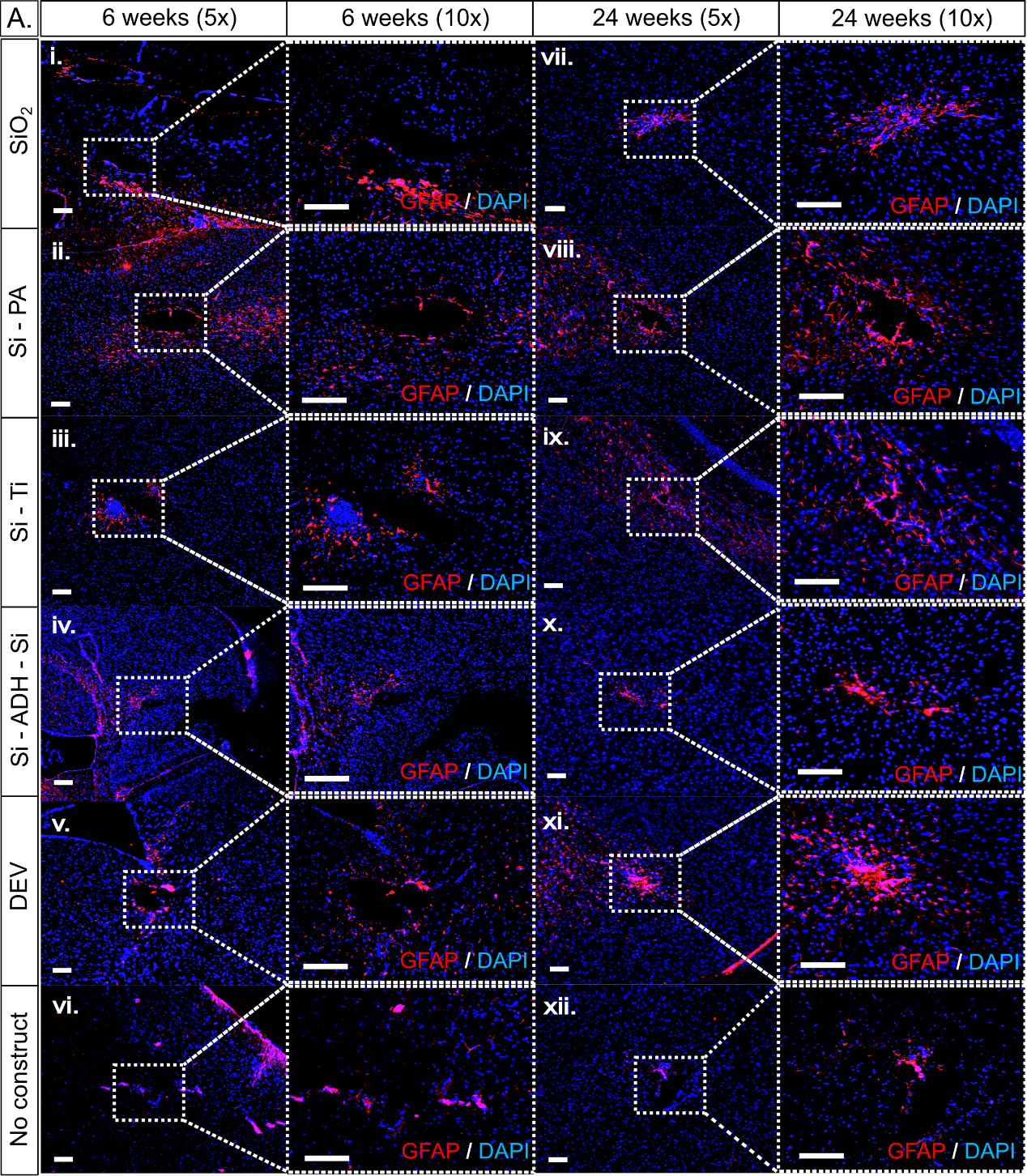


**Figure S2.** Fluorescent images displaying cells stained with GFAP at 6 (i-vi) & 24 weeks (vii-xii) post-implantation of the microdevices. Included in this figure are all the other microconstructs: silicon oxide (i & vii), silicon coated with a PA layer (ii & viii), silicon with one-side titanium coating (iii-ix), two silicon parts glued with UV curing adhesive (iv & x), silicon with one-side coated with UV curing adhesive and the second-side coated with silicon oxide plus a titanium layer (v & xi), and a blank sample with no construct (vi & xii). GFAP staining reveals astrocyte accumulation by each microconstruct and subsequent glial scar formation. Mouse brains implanted with the remaining silicon-based microdevices exhibited low levels of astrocyte accumulation comparable to LATEX (scale bars = 100 μm). The graph illustrating glial scar formation and the assessment of astrocyte numbers is presented in fig. 6B of the main text, highlighting LATEX as displaying the highest number of GFAP+ cells at both 6 & 24 weeks post-implantation.


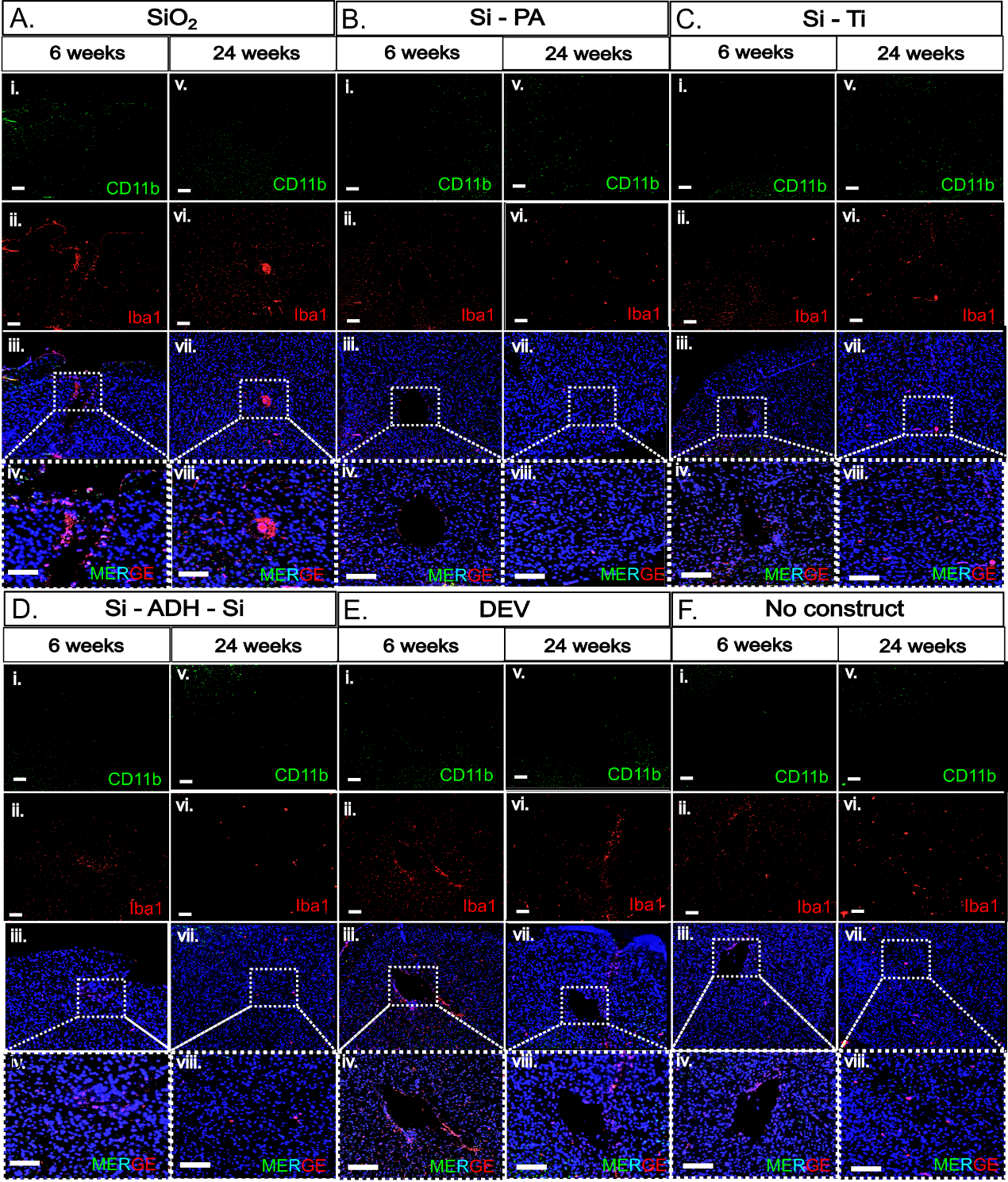


**Figure S3.** Fluorescent images depicting cells stained with CD11b & Iba1 at 24 weeks post-implantation of the microconstructs. All other microconstructs are included in this figure: silicon oxide (A), silicon coated with a PA layer (B), silicon with one-side titanium coating (C), two silicon parts glued with UV curing adhesive (D), silicon with one-side coated with UV curing adhesive and the second-side coated with silicon oxide plus a titanium layer (E), and a blank sample with no construct (F). Similar to previous observations, mouse brains stained with the astroglial/macrophage marker CD11b displayed consistent numbers of CD11b+ macrophages at both 6 weeks (i-iv) and 24 weeks (v-viii) after implantation for all conditions. Separate channel images for CD11b (5x) (i & v), Iba1 (5x) (ii & vi), and merged CD11b/Iba1 (5x) (iii & vii), as well as 10x MERGE (10x) (iv & viii) are presented. **As depicted in Figure 5E in the main text**, LATEX exhibited a similar number of CD11b+ cells at both 6 & 24 weeks post-implantation compared to all other conditions. Furthermore, mouse brains stained with the microglial marker Iba1 demonstrated elevated expression of Iba1+ macrophages at 6 & 24 weeks post-implantation without significant differences between the two timepoints for the same construct. Fluorescent images of mouse brains stained with the microglial/macrophage marker Iba1 revealed a 3-fold increase in the number of Iba1+ macrophages in brains implanted with LATEX compared to silicon or HDPE (i-xi) (scale bars = 100 μm).
